# Parity and Risk of Coronary Heart Disease in Middle-aged and Older Chinese Women

**DOI:** 10.1038/srep16834

**Published:** 2015-11-26

**Authors:** Lijun Shen, Jing Wu, Guiqiang Xu, Lulu Song, Siyi Yang, Jing Yuan, Yuan Liang, Youjie Wang

**Affiliations:** 1 MOE Key Lab of Environment and Health, School of Public Health, Tongji Medical College, Huazhong University of Science & Technology, Wuhan, 430030, China; 2 Department of Maternal and Child Health, School of Public Health, Tongji Medical College, Huazhong University of Science & Technology, Wuhan, 430030, China; 3 Department of Social Medicine, School of Public Health, Tongji Medical College, Huazhong University of Science & Technology, Wuhan, 430030, China

## Abstract

Pregnancy leads to physiological changes in lipid, glucose levels, and weight, which may increase the risk of coronary heart disease (CHD) in later life. The purpose of this study was to examine whether parity is associated with CHD in middle-aged and older Chinese women. A total of 20,207 women aged 37 to 94 years from Dongfeng-Tongji Cohort who completed the questionnaire, were medically examined and provided blood samples, were included in our analysis. CHD cases were determined by self-report of physician diagnosis through face-to-face interviews. Logistic regression models were used to estimate the association between parity and CHD. The rate of CHD was 15.8%. Parity had a positive association with CHD without adjustment of covariates. After controlling for the potential confounders, increasing risk of coronary heart disease was observed in women who had two (OR, 1.65; 95% CI, 1.41–1.93), three (OR, 1.76; 95% CI, 1.44–2.16), and four or more live births (OR, 1.71; 95% CI, 1.33–2.20) compared with women with just one live birth. High parity was significantly associated with increasing risk of CHD in Chinese women. This suggests that multiparity may be a risk factor for CHD among Chinese women.

Cardiovascular disease (CVD) is responsible for 30% of all deaths worldwide^1^. Coronary heart disease (CHD) as the predominant form of CVD, is the leading cause of death in Western countries and United States from CVD^2^,^3^. China is also challenged by the epidemic of CVD with approximately 32% of total deaths in 2005^4^. It is projected that the CVD incidence and mortality will increase substantially during the next 20 years^5^. A set of risk factors have been identified as contributing to the occurrence of CHD, such as smoking, elevated blood pressure, diabetes and obesity^6^. In addition to these factors, which have similar impacts in both men and women, it is believed that reproductive history plays an important role in the development of CHD in middle-aged and older women.

Pregnancy leads to a series of physiological changes. During normal pregnancy, insulin level increases under the influence of gestational hormones. Insulin resistance increases, and β-cells proliferate to maintain maternal euglycemia^7^. Lipid levels also rise, except for high density lipoprotein (HDL), which rises progressively during the first two trimesters and then falls during the third^8^,^9^. Changes in insulin and lipid concentration may have an adverse effect in later life^10^,^11^. Child-rearing may also adversely affect women, because of the stress and anxiety of parenting^12^. Both the physiological and lifestyle changes may suggest a potential mechanism for the occurrence of CHD.

The association of parity and CHD has been described in several studies, but the results are inconsistent^13^,^14^,^15^,^16^. Most found that women with more children were at a higher risk of CHD than nulliparous women^14^,^15^. The latest study found a “J” shaped association between number of children and CHD prevalence, after adjustment for other confounding factors^17^. In contrast to these findings, there are also reports of a negative association between parity and CHD^13^,^16^.

Studies about the association between parity and CHD have been conducted in women of several different nationalities, but none have previously studied Chinese women. To explore whether the association exists in Chinese women, because of the difference of race, culture and lifestyle, we evaluated the association between parity and CHD in Chinese middle-aged and older women.

## Results

### Association between parity and CHD risk factors

Of the 20,207 women, 3,183 (15.8%) had CHD. The age-adjusted relationship between parity and CHD risk factors are shown in Table 1. Older women tended to have had more births than younger ones. Parity was significantly associated with education, marital status, and occupation (all *P* < 0.001). Women with higher parity tended to have lower education levels, be divorced or widowed, and to be manual occupation. Higher parity was significantly associated with smoking, drinking and inactivity (all *P* < 0.001). BMI, WHR, and SBP increased significantly with parity (all *P* < 0.001). Levels of HDLc and total cholesterol were negatively associated, and glucose was positively associated, with parity (all *P* < 0.001). No linear relationships were found between parity and DBP, triglycerides and LDLc. Women with higher parity were more likely to have a history of stillbirth and less likely to be postmenopausal and to have contraceptive and HRT use (all *P* < 0.001). The prevalence of hypertension and diabetes were higher in the multiparous groups.

### Association between parity and CHD

**Figure 1:**
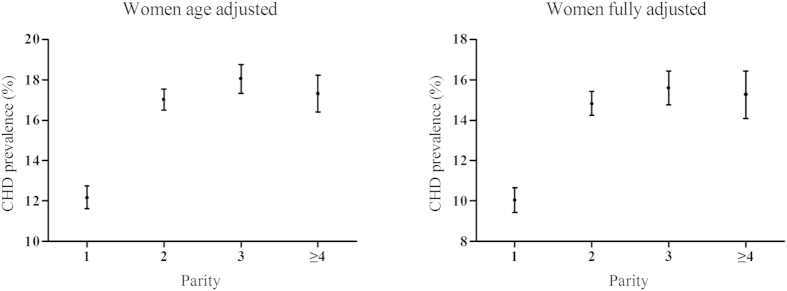
Age-adjusted and fully-adjusted CHD prevalence (95% confidence interval) by parity in Chinese women.

The age-adjusted and fully adjusted prevalence of CHD by parity are shown in Fig. 1. There was an upward trend between parity and CHD prevalence, with the lowest prevalence among those with one live birth. Both the unadjusted and multivariable models showed that greater parity was significantly associated with an increasing risk of CHD, as shown in Table 2. After adjusting for demographic, lifestyle, reproductive and known mediating factors, ORs were 1.65 (95% CI: 1.41–1.93), 1.76 (95% CI: 1.44–2.16), and 1.71 (95% CI: 1.33–2.20) for women with two, three, and four or more live births.

## Discussion

To our knowledge, this is the first study assessing the association between parity and the risk of CHD in Chinese women. Our results indicate that parity may increase the risk of CHD, since the prevalences of CHD were found to be higher in multiparous women than in those with only one live birth. Even after accounting for age, lifestyle, metabolic and other potential confounding factors, the positive association did not alter.

Our findings are consistent with previous studies that have shown that parity increases the risk of CHD. For example, Ness *et al.* analyzed two cohorts of 2,357 and 2,533 women and showed that the women who had six or more pregnancies had an increased risk of CHD in both cohorts, after adjusting for age, educational level, BMI and other CHD risk factors^14^. A cross-sectional study enrolled 64,530 US black women and found a positive association between higher parity (≥7) and increased risk of CHD^15^. These reports, however, only found that women with more than five live births had an increased risk of CHD. Our study found this increased risk in every category compared with one live birth. Lawlor *et al.* conducted a study of 4,286 women and 4,252 men aged 60–79 years. They found a “J” shaped association between number of children and CHD in both men and women^17^. In our study, by contrast, the “J” shaped association was not specific, because the odds ratio of the parity above four decreased slightly compared with other groups. Cardiovascular disease is the largest cause of death in middle aged and older women in China^18^. It is likely that some older women died of CHD before our study. If these individuals had high parity, in line with the data observed in the study, this would lead to an underestimation of the risk because of the survivor bias. A prospective study showed that high gravidity (≥4) reduced the risk of cardiovascular mortality^19^. These inconsistent results may be due to the difference of study design, sample size, and demographic characters. More studies are needed to verify the effects of parity on CHD.

The mechanism of the association of parity and CHD is not well understood. Pregnant women are in a state of insulin resistance. Repeated pregnancies might aggravate the effects on glucose, and these effects might persist for decades, leading to diabetes in the future^20^,^21^,^22^. In addition, HDLc would decline after the first birth^23^. Parity may also aggravate the effect on the lower level of HDLc in multiparous than in nulliparous women^11^,^23^,^24^,^25^. More importantly, weight gain during pregnancy is associated with long-term weight^26^. Women with higher parity are more likely to be overweight or obese, and have excess deposition of intra-abdominal and visceral fat^27^,^28^,^29^,^30^,^31^. Since diabetes, low HDLc and obesity are risk factors for CHD, these metabolic mechanisms may partly explain the association between parity and CHD.

Estrogen is considered as a protective factor against the developing of CHD. During normal pregnancy, the secretion of estrogen is interrupted along with the reset of ovarian function. Greater parity could influence the estrogen concentrations with pregnancy and lactation which reduces the lifetime exposure on endogenous estrogens^32^. Parity is also reported to be associated with carotid artery atherosclerosis and carotid artery distensibility in elderly women^33^,^34^. These effects may play an important role in the association of parity with the risk of CHD.

Several studies which made a comparison of women and men have shown that childrearing related factors were related to the increase of cardiovascular events^17^,^35^. Higher parity, for example, may lead to unhealthier behaviors, such as smoking and alcohol consumption, because of the higher levels of stress associated. Socio-economic factors may also have an effect on parity. However, these factors are difficult to measure or fully include in studies. In the present study, we could only take into consideration education, marriage status, occupation, and lifestyle factors including cigarette smoking, passive smoking, alcohol drinking, and physical activity, none of which eliminated the significant association. Only passive smoking was found to be significantly related to CHD in the fully adjusted model.

Our study has some important strengths, in particular the large sample size. We also included abundant potential confounders in our study, which may not have been considered adequately in previous studies. Finally, the study took place in hospitals. The use of trained health professionals, standardized questionnaires and medical examinations make the data more reliable.

There are also some limitations to our study. First, it was a cross-sectional study, so we could not determine the causal relationship between parity and CHD. It is, however, considered unlikely that women with CHD tend to have more pregnancies. CHD is also a chronic disease among older people. We considered that they had probably completed reproduction before developing CHD. Second, CHD and parity were obtained from self-reporting, which could be affected by information bias. People with CHD may not have recognized their illness causing under-reporting of disease levels. Thirdly, there are some potential confounders we did not measure. Women with pregnancy complications, such as gestational diabetes and pregnancy-induced hypertension, are reported to have higher levels of CHD in later life^36^. Another limitation of the study is that our findings were restricted to retirees. Working women have been found to have more favorable CHD risk factors than women who stayed home^37^,^38^. Finally, because of the differences of regions and cultures, the women in our study may not be representative of Chinese women more generally.

In summary, our study suggested that parity is associated with increased risk of CHD. Biological effects may play an important role in the association. Since the mechanism that accounts for the association is unclear, further investigation is needed to assess the independent effect of biological, socio-economic and lifestyle factors on risk of developing CHD, alongside childbirth and child-rearing.

## Methods

### Study participants

The study subjects were from the Dongfeng-Tongji Cohort study phase II. The design and method of this study have been described elsewhere^39^. Phase II was launched in 2013 and recruited 38,295 participants, who agreed to answer the questionnaires and receive a medical examination. A total of 21,187 women were enrolled in our study. We excluded women whose information on CHD and parity were missing. Since women may be childless as a result of health- related factors such as infertility, we excluded any participants who had no live birth to avoid potential bias. The total excluded was 980 women (2.7% of the population), and 20,207 participants were therefore included in the analysis.

The study was approved by the Medical Ethics committee of the School of Public Health, Tongji Medical College, and Dongfeng General Hospital. Informed consent was obtained from all participants. The study was performed according to the approved guidelines.

### Assessment of CHD and parity

CHD in our study was determined by self-reporting of a physician diagnosis of CHD and myocardial infarction (MI). Participants were asked the questions “Have you ever been diagnosed with CHD” or “Have you ever been diagnosed with MI”. The answer of “yes” was defined as the CHD case. Parity was defined as self-reporting of the number of live births.

### Assessment of covariates

Standing height, weight, waist circumference, seated blood pressure, total cholesterol, high-density lipoprotein cholesterol (HDLc), low-density lipoprotein cholesterol (LDLc), triglycerides (TG), and fasting plasma glucose were obtained from the medical examination. Body mass index (BMI) was defined as weight (kg)/height^2^ (m^2^). Waist to hip ratio (WHR) was defined as waist circumference/hip circumference. Age, marital status (e.g., married, single, divorced, widowed), education levels (e.g., primary or below, junior high school, high school, college or above) and the occupation before retirement were collected via questionnaire, as well as lifestyle and reproductive information. The occupations were classified into two categories (manual job or non-manual job). Physical activity was defined as doing exercise more than three times a week for more than 20 minutes. Current smoking was defined as smoking at least once a day over a period of more than half a year. Current drinking was defined as drinking once a week for a period of more than half a year. Menopause was defined as ceasing menstruating at least one year. Diabetes was defined as fasting plasma glucose (FPG) level ≥7.0 mmol/L, a self-reported physician diagnosis of diabetes, or use of hypoglycemic agents or insulin^40^. Hypertension was defined as a self-reported physician diagnosis of hypertension, antihypertensive use, or systolic blood pressure (SBP) ≥ 140 mmHg or diastolic blood pressure (DBP) ≥90mmHg^41^.

### Statistical analysis

Analysis of covariance (ANCOVA) and linear regression were used to assess the age-adjusted means and linear trend for continuous variables. Logistic regression was used to assess the age-adjusted prevalence for categorical variables. The regression coefficient for continuous variables and odds ratios for categorical variables were used to describe the difference with each additional birth. The age-adjusted and fully adjusted prevalence of CHD by parity was assessed graphically. A series of multiple logistic regression analyses were performed to calculate the odds ratio (OR) and 95% confidence intervals (95% CI) of the association of the risk of CHD with parity categories. The women with one live birth were used as the reference group. We adjusted age in model 1, age, demographic factors and lifestyle (marital status, education, occupation, family history of CHD, smoking, passive smoking, drinking and physical activity) in model 2, and age, demographic factors, lifestyle and reproductive factors (abortion, stillbirth, menopause status, hormone replacement therapy (HRT), and contraceptive use) in model 3. Finally, we adjusted age, demographic factors, lifestyle, reproductive factors and mediating factors reported as CHD risk factors (total cholesterol, HDLc, LDLc, triglycerides, BMI, WHR, hypertension, diabetes) in model 4. All the statistical analyses were performed using SAS software (version 9.4; SAS Institute Inc, Cary, NC, USA). Statistical significance was defined as *P* < 0.05.

## Additional Information

**How to cite this article**: Shen, L. *et al.* Parity and Risk of Coronary Heart Disease in Middle-aged and Older Chinese Women. *Sci. Rep.* **5**, 16834; doi: 10.1038/srep16834 (2015).

## Figures and Tables

**Table 1:** Age-adjusted means and prevalence of CHD risk factors by parity in Chinese women.

	Parity				Difference per increase of 1 birth^a^	*P* for trend
	1(N = 9283)	2(N = 5906)	3(N = 3158)	≥4(N = 1860)		
Age (years)	55.83 (55.71, 55.96)	64.10 (63.94, 64.25)	69.86 (69.65, 70.07)	74.53 (74.26, 74.80)	6.59 (6.50, 6.67)	0.000
BMI (kg/m^2^)	23.75 (23.66, 23.84)	24.41 (24.32, 24.51)	25.00 (24.85, 25.14)	24.99 (24.78, 25.19)	0.48 (0.41, 0.56)	0.000
WHR	0.853 (0.851, 0.855)	0.865 (0.863, 0.868)	0.874 (0.870, 0.877)	0.877 (0.872, 0.882)	0.009 (0.007, 0.010)	0.000
SBP (mmHg)	135.58 (135.01, 136.15)	137.90 (137.31, 138.50)	138.87 (138.96, 140.79)	138.11 (136.83, 139.38)	1.28 (0.80, 1.76)	0.000
DBP (mmHg)	77.58 (77.26, 77.90)	78.37 (78.04, 78.70)	78.78 (78.27, 79.29)	77.81 (77.10, 78.52)	0.25 (–0.02, 0.52)	0.066
Triglycerides (mmol/L)	1.56 (1.54, 1.59)	1.58 (1.55, 1.61)	1.55 (1.50, 1.60)	1.47 (1.41, 1.54)	–0.023 (–0.047, 0.001)	0.061
LDLc (mmol/L)	2.80 (2.78, 2.83)	2.78 (2.75, 2.80)	2.81 (2.78, 2.85)	2.78 (2.73, 2.83)	–0.003 (–0.022, 0.016)	0.764
HDLc (mmol/L)	1.61 (1.60, 1.62)	1.54 (1.53, 1.56)	1.49 (1.47, 1.51)	1.45 (1.42, 1.47)	–0.05 (–0.06, –0.04)	0.000
Total cholesterol (mmol/L)	5.03 (5.00, 5.06)	4.91 (4.88, 5.94)	4.84 (4.80, 4.89)	4.75 (4.68, 4.81)	–0.09 (–0.12, –0.07)	0.000
Glucose (mmol/L)	5.84 (5.79, 5.88)	5.99 (5.95, 6.04)	6.05 (5.99, 6.12)	6.15 (6.06, 6.25)	0.11 (0.07, 0.14)	0.000
Education (>primary, %)	92.78 (92.12, 94.43)	76.71 (75.65, 77.78)	62.05 (60.11, 64.00)	41.07 (38.13, 44.01)	0.40 (0.38, 0.42)	0.000
Divorced/widowed (%)	15.12 (14.09, 16.18)	14.40 (13.56, 15.24)	15.45 (14.29, 16.61)	20.54 (18.73, 22.35)	1.13 (1.07, 1.20)	0.000
Manual occupation (%)	52.31 (51.11, 53.51)	67.46 (66.36, 68.56)	81.67 (80.48, 82.86)	91.67 (90.71, 92.63)	2.26 (2.14, 2.37)	0.000
Current smoking (%)	1.26 (1.00, 1.53)	1.62 (1.30, 1.95)	3.15 (2.44, 3.86)	4.86 (3.52, 6.19)	1.63 (1.43, 1.87)	0.000
Passive smoking (%)	32.60 (31.47, 33.72)	29.09 (27.88, 30.29)	28.29 (26.41, 30.16)	29.84 (27.16, 32.51)	0.93 (0.89, 0.98)	0.005
Current Drinking (%)	9.40 (8.80, 9.94)	11.64 (10.74, 12.55)	13.89 (12.20, 15.58)	15.54 (12.89, 18.19)	1.24 (1.15, 1.33)	0.000
Physical activity (%)	74.30 (73.22, 75.39)	75.32 (74.20, 76.44)	73.90 (72.17, 75.64)	66.29 (63.67, 68.91)	0.91 (0.87, 0.95)	0.000
Menopause (%)	90.62 (90.25, 90.98)	90.76 (89.82, 91.69)	89.54 (86.77, 92.31)	71.17 (66.49, 75.86)	0.78 (0.69, 0.89)	0.000
Ever use contraceptive (%)	19.06 (18.07, 20.06)	19.71 (18.67, 20.75)	16.83 (15.36, 18.30)	10.79 (9.19, 12.39)	0.85 (0.81, 0.90)	0.000
Ever use HRT (%)	3.83 (3.31, 4.35)	2.74 (2.32, 3.16)	1.89 (1.38, 2.40)	0.88 (0.44, 1.32)	0.66 (0.57, 0.75)	0.000
Abortion (%)	74.61 (73.50, 75.73)	65.80 (64.57, 67.04)	60.63 (58.69, 62.57)	52.12 (49.33, 54.91)	0.72 (0.69, 0.76)	0.000
Stillbirth (%)	1.26 (1.01,1.51)	2.48 (2.06,2.91)	2.39 (1.73,3.04)	3.29 (2.15,4.43)	1.34 (1.16,1.55)	0.000
Family history of CHD (%)	20.46 (19.29, 21.64)	11.22 (10.32, 12.12)	7.12 (6.06, 8.18)	5.41 (4.18, 6.64)	0.57 (0.52, 0.61)	0.000
Hypertension (%)	56.48 (55.34, 57.62)	59.47 (58.22, 60.72)	62.47 (60.45, 64.49)	60.02 (57.06, 62.99)	1.10 (1.05, 1.15)	0.000
Diabetes (%)	16.23 (15.21, 17.24)	21.69 (20.61, 22.77)	24.83 (23.16, 26.50)	25.38 (23.07, 27.70)	1.22 (1.16, 1.29)	0.000

Values are means and prevalences (95% confidence intervals). All means, prevalences, regression coefficients and odds ratios are age-adjusted, except for age.

^a^For continuous variables, regression coefficients of unit increase in the variables per increase of one birth, for dichotomous variables, odds ratio for increase in one birth.

**Table 2:** Unadjusted and adjusted odd ratio (95% CI) for CHD by parity.

	Parity			
	1	2	3	≥4
No.of subjects	9283	5906	3158	1860
No.of cases	689	1069	831	594
Unadjusted model	1	2.76 (2.49, 3.05)	4.46 (3.99, 4.98)	5.86 (5.17, 6.63)
Model1	1	1.51 (1.35, 1.70)	1.63 (1.42, 1.88)	1.55 (1.31, 1.84)
Model2	1	1.87 (1.63, 2.15)	2.13 (1.78, 2.54)	2.00 (1.61, 2.48)
Model3	1	1.85 (1.60, 2.14)	2.11 (1.76, 2.53)	2.05 (1.64, 2.57)
Model4	1	1.65 (1.41, 1.93)	1.76 (1.44, 2.16)	1.71 (1.33, 2.20)

Model 1: age;

Model 2: model 1 + marital status, education, occupation, history of CHD, smoking, passive smoking, drinking, physical activity;

Model 3: model 2 + abortion, stillbirth, menopause status, contraceptive use, hormone replacement therapy;

Model 4: model 3 + Total cholesterol, HDLc, LDLc, triglycerides, BMI, WHR, hypertension, diabetes.
